# Urgent Versus Delayed Surgical Treatment of Open Distal Radius Fractures: A Multicenter Retrospective Study

**DOI:** 10.1016/j.jhsg.2025.03.006

**Published:** 2025-05-22

**Authors:** Luke Latario, Matthew DeFazio, Matthew Poorman, Alan Shi, Eric Swart, Marci Jones

**Affiliations:** ∗ Department of Orthopedic Surgery, University of Massachusetts Memorial Medical Center, Worcester, MA; † Department of Orthopedic Surgery, Lahey Hospital and Medical Center, Burlington, MA

**Keywords:** Hand, Infection, Surgery, Fracture

## Abstract

**Purpose:**

Controversy exists on the urgency of operative treatment for low energy open distal radius fractures. Two medical centers shifted practice and no longer take all open distal radius fractures emergently to the operating for debridement. The purpose of this study is to provide preliminary data to evaluate if this is associated with unacceptably high infection rates.

**Methods:**

A retrospective chart review was performed for 55 open distal radius fractures in 54 patients at two level one trauma centers. Patients underwent irrigation and closed reduction in the emergency department followed by definitive closed treatment or operative treatment with surgical irrigation, debridement, and fixation, either within 24 hours or after discharge. The main outcome measure was infection, with secondary outcomes of complications or secondary procedures.

**Results:**

In this cohort, 4 of 55 patients experienced infections (7.2%.) There were no infections in low energy injuries. Twenty-four patients went to the operating room 2 or more days from presentation or were treated nonoperatively with only 1 postoperative infection (4.2%). All infections were in high energy mechanism injuries with at least one additional risk factor: smoking, polytrauma, or Gustilo Anderson type 2 injury.

**Conclusions:**

These data suggest that in patients with low energy injury mechanisms and type 1 injuries, delay in formal operative debridement is not associated with an elevated infection risk in this small case series. Future prospective studies with larger sample sizes are needed to definitively evaluate whether some open distal radius fracture patterns may not require emergent operative debridement.

**Type of study/level of evidence:**

Prognostic IV.

Distal radius and ulna fractures are fractures of the wrist involving the metaphyseal bone of either the radius alone, or the radius and ulnar styloid, and are a common injury. With an annual incidence of between 57–100 per 10,000 in the United States, they have a particularly high prevalence and large morbidity burden in older osteoporotic patients. It is estimated that 2% to 6% of distal radius fractures are open injuries.^1^^,^^2^ While the risk of infection for open fractures of the hand is low compared to that of long bones and the lower extremity, the incidence of infection in open distal radius fractures is reportedly between 5% to 11%.^3^, ^4^, ^5^, ^6^

Antibiotics are well established in reducing the rate of infection in open fractures of the extremities.^5^^,^^7^ However, the duration of time from injury to administration of antibiotics to reduce incidence of infection for open distal radius fractures has not been established. Zumsteg et al^6^ were not able to correlate the average time to antibiotics with infection rate in a study of 200 open forearm fractures. Based on the relative safety of antibiotics and their demonstrated benefit, initiating antibiotic therapy as early as possible often is recommended in open distal radius fractures.^8^

Additionally, early operative irrigation and debridement for open fractures is a strongly recommended principle in orthopedics to minimize the rate of infection.^5^ However, the specific timing of debridement is controversial based on the extent of contamination, soft tissue injury and specific anatomic location.^4^^,^^5^^,^^9^, ^10^, ^11^ Some investigators advocate immediate debridement of open distal radius fractures even in low energy fragility fracture.^10^ Other work has demonstrated similar functional outcomes in type 1 open distal radius fractures between those treated urgently and those in a delayed fashion.^9^^,^^12^ Kurylo et al^12^ in a cohort of 32 open distal radius fractures, found no increased infection risk for fractures debrided in <6 hours compared to those debrided after 6 hours, with a mean time to the operating room of 20 hours. Henry et al^9^ evaluated a cohort of 24 patients with type 1 open fractures and found no difference in functional outcomes between the 17 patients who underwent surgery within 24 hours and the 7 patients who underwent surgery >24 hours after injury. Other work suggests that the grade of soft tissue injury and level of gross contamination are the main risk factors determining the rate of infection, and that the time to antibiotics and operating room are not considerable factors in cases with debridement and fixation within 24 hours.^3^^,^^6^^,^^8^^,^^13^

A paucity of high-level evidence exists regarding optimal timing of surgical treatment for open distal radius fractures and the possible association with postoperative complication and infection, especially in those undergoing surgical treatment >24 hours from injury. Debate remains regarding the relative urgency in the time to debridement and definitive fixation for open distal radius fractures. Newer studies further suggest that some open distal radius fractures can be appropriately triaged to outpatient surgery without an increased rate of infection or complications.^14^^,^^15^

At the level 1 trauma centers in this study, we do not routinely take low-grade open fractures for emergent irrigation and debridement. Many patients are discharged after bedside irrigation and reduction for further treatment at ambulatory centers. We performed a retrospective review of patients with open distal radius and ulna fractures treated with antibiotics and irrigation and debridement at 2 tertiary care facilities to evaluate the effect of this practice on infection rate. The purpose of this study is to evaluate whether delay in definitive debridement or fixation of open distal radius fractures is associated with higher infection or complication rate compared to historic infection rates reported for urgent or emergent irrigation and debridement. Given the experience at this study’s institutions, along with recent trends in the literature, we hypothesized that there would be no increased rate of infection in patients who had delayed definitive debridement and fixation of their open distal radius fractures.

## Materials and Methods

After institutional review board approval and data use agreement between medical centers, a retrospective chart review was performed of open distal radius fractures at 2 level I trauma centers. Database was queried for AO/OTA fracture type 23R and 23U. Electronic medical records were reviewed by 1 of 3 trained hand surgery residents and 1 trained research assistant. Inclusion criteria were all open distal radius fractures in adult patients evaluated in the emergency department by an hand surgery resident between January 1, 2015, and December 31, 2020. Patients <18 years old were excluded from this study.

Patient demographic factors were collected including age, sex, smoking status, and presence of diabetes. Sex was recorded as the male or female assigned sex at birth as reported in the electronic medical record. Patient time to debridement, antibiotics, and mechanism of injury also were recorded. Complications were defined broadly as unplanned adverse medical events related to the distal radius injury or treatment. This would include, for example, wound infection, acute carpal tunnel syndrome, or return to the emergency department for uncontrolled pain. Falls from standing were categorized as low energy.

Gustilo wound classification was determined from the orthopedic consultant resident’s documentation and attending surgeon’s operative notes if applicable. Superficial abrasions were not classified as open injuries. Wounds <1 cm were classified as type 1 injuries, wounds >1 cm but <10 cm were type 2, wounds >10 cm or having considerable bone loss or soft tissue injury requiring local or flee flap coverage were classified as type 3 injuries.^16^^,^^17^ Falls from standing were categorized as low energy. Falls from any height greater than standing, and motor vehicle collisions were classified as high energy. Clinical outcomes including the presence of postoperative infection, duration of follow-up, postoperative complication including readmissions, and return to the emergency department also were reviewed. Postoperative infection was assessed case by case via chart review, to identify patients who either received an unplanned course of antibiotics after initial surgery or patients who returned to the operating room for unplanned repeat irrigation and debridement secondary to concern for infection.

At these two institutions, the general practice for management of open fractures falls into 1 of 2 categories. The first are patients with high-grade, high energy injury mechanisms, or polytraumatized patients, who are given with antibiotics and urgent operating room debridement and fixation; and then lower acuity patients with low-grade, low energy fractures, who are treated with urgent antibiotics and bedside irrigation, consisting of low-pressure superficial wash using normal saline or diluted betadine solution in the emergency department without extension of traumatic wounds. This is followed by provisional wound closure if required using nylon suture and reduction with splinting or casting. In those cases, the decision for further surgery is based on fracture pattern and alignment in discussion with patient’s preferences, rather than the presence of an open injury.

## Results

Between January 1, 2015, and December 31, 2020, 64 AO/OTA type 23R open distal radius fractures in 63 patients were evaluated and underwent closed reduction and splinting. One patient sustained bilateral open distal radius injuries. All patients received antibiotics in the emergency department on presentation, with the exception of 1 patient who had their open fracture diagnosed at the time of fixation 12 days after injury. This patient did not experience an infection. Of the 63 patients treated for open distal radius injuries, 9 were lost to follow-up and did not undergo definitive treatment with either closed management or subsequent irrigation and debridement with definitive fixation and, therefore, were excluded. This left a cohort of 55 injuries in 54 patients. Fifty patients received antibiotics the day of their injury, and 5 patients were administered antibiotics 1 day after their injury because of delay in presentation or evaluation. None of the 5 patients prescribed antibiotics with a delay of 1 day from their date of injury had an infection. Mean and median follow-up was 33 and 16 weeks, respectively, with a range of 3.4 weeks to 4.8 years.

Three patients were treated definitively with closed reduction and splinting with follow-up to fracture union. None of these experienced infections and all were treated successfully nonoperatively to union. These patients were included within the group of ≥2 days to the operating room.

Among all operatively treated patients, the average time to the OR from presentation was 2.3 days (Table 1). Among all patients (including those treated nonoperatively), there were 4 infections (7.2%) and an overall complication rate of 31% (Table 1, Table 2). The most frequent complications were infection that required return to the operating room for additional irrigation and debridement (*n* = 4), nonunion (*n* = 2), neurapraxia (*n* = 5), and return to the emergency department (*n* = 2.) Twenty-three patients underwent a second procedure. The most common reasons for reoperations were for symptomatic retained implant (*n* = 3) or as part of a staged procedure, such as external fixation (*n* = 9), or dorsal spanning plate (*n* = 11).

**Table 1:** Patient Demographics

Characteristics and Outcomes	All Open Fractures	High Energy	Low Energy	Patients Discharged Without Operative Irrigation and Debridement
Total number of patients	55	28	27	24
Mean age (y)	60	51	69	66
Number of male	21 (38%)	18 (64%)	3 (11%)	10 (42%)
Number of female	34 (61%)	10 (36%)	24 (89%)	14 (58%)
Number of active smokers	21 (38%)	15 (54%)	6 (22%)	7 (29%)
Number diabetic	3 (7.8%)	3 (11%)	0	2 (8%)
Number of patients sustaining other injuries	32 (58%)	21 (75%)	11 (41%)	8 (33%)
Number of Gustilo Anderson Type 1	29 (52%)	14 (50%)	15 (56%)	15 (62%)
Number of Gustilo Anderson Type 2	23 (42%)	11 (39%)	12 (44%)	9 (38%)
Number of Gustilo Anderson Type 3	3 (5.5%)	3 (11%)	0	0
Number undergoing operative treatment ≤1 d from injury	31 (56%)	19 (68%)	12 (44%)	2 (8%)
Number undergoing operative treatment >2 d from injury & nonsurgical treatment	24 (44%)	9 (32%)	15 (55%)	22 (92%)
Mean time to debridement (d)	2.3	1.4	3.2	4.4
Number undergoing a second surgery	23 (42%)	16 (57%)	7 (26%)	6 (25%)
Number experiencing a complication	17 (31%)	10 (36%)	7 (26%)	6 (25%)
Number of infections	4 (7.2%)	4 (14%)	0	1 (4.2%)

Parentheses denotes percent group or subgroup.

**Table 2:** Patients Who Experienced Postoperative Infections

Characteristics and Outcomes	Patient 1	Patient 2	Patient 3	Patient 4	Mean
Age (y)	50	53	31	75	52
Sex	Male	Male	Male	Female	-
Smoking	No	No	Yes	Yes	-
Diabetic	No	No	No	No	-
Mechanism	Fall from height	Fall from ladder	Dirt motor bike	Fall down stairs	-
Other injuries	None	Elbow fracture dislocation	Facial bone fractures, bilateral open distal radius fractures, bowel perforation	None	-
Gustilo Anderson Type	2	2	3	1	2
Discharged for treatment at ambulatory center	No	No	No	Yes	-
Time to debridement (d)	0	1	1	2	1
Additional surgery	Irrigation & debridement, revision fixation, proximal row carpectomy	Irrigation & debridement, revision fixation, bone grafting	Irrigation & debridement, revision fixation, skin grafting	Irrigation & debridement, removal of implant	
Complication	Malunion, posttraumatic osteoarthritis	Delayed union	Osteomyelitis	Infected nonunion	

Twenty-four patients went to the operating room ≥2 days after injury or were treated nonoperatively. Mean time to the OR for these ambulatory patients was 4.4 days. Only 1 (4.2%) infection developed among the 24 outpatient surgeries, and none of the nonsurgical patients had an infection. The remaining 31 patients underwent irrigation and debridement within 1 day of presentation and 3 of these experienced infection (9.7%) (Table 1).

Twenty-eight patients sustained a high energy mechanism of a fall from height greater than standing, or motor vehicle collisions, and 27 sustained low energy mechanisms (Table 1). Patients who sustained high energy injuries were on average treated with surgical irrigation and debridement 1.4 days from injury and those with low energy injuries 3.2 days. There was a higher reoperation rate (57%) and overall complication rate (36%) in patients with high energy mechanisms when compared to the low energy group, which had a reoperation and complication rate both of 26%.

Overall, 4 patients experienced infections (Table 2). Of these, 3 patients had urgent operative irrigation and debridement within 1 day of presentation. Only 1 patient who later had an infection underwent irrigation and debridement 2 days after injury and was treated at an ambulatory day surgery center after being discharged from the emergency department. All patients with infections sustained a high energy mechanism of injury and possessed at least 1 additional risk factor for infection, such as active smoking, polytrauma, or Gustilo Anderson type ≥2 soft tissue injury (Table 2).

## Discussion

Open fractures result in soft tissue disruption and possible contamination of bone with infectious debris. Subsequently, orthopedic surgical management of open long bone fractures has emphasized early debridement, irrigation, and antibiotics.^5^ However, in the upper extremity there is a lower incidence of infection after open fracture, and deep infection often is attributed to gross contamination in larger wounds.^9^^,^^13^ Zumsteg et al^6^ in a retrospective series of 200 open forearm fractures, found no benefit in decreased deep infection or union rate among patients with open fractures treated within the historic limit of 6 hours. Other studies of only open distal radius fractures similarly have shown no benefit in operative treatment fractures in less than 6 hours.^12^ However, considerable debate remains on the safe duration of delay for operative treatment beyond 24 hours, and some investigators advocate early debridement.^1^^,^^4^^,^^10^

In our retrospective study, there were no infections in low energy injuries and a 14% infection rate in patients with high energy mechanisms of injury. Patients who experienced infections in our cohort carried additional risk factors for infection, such as type >1 Gustilo Anderson soft tissue injury or active smoking. Among the low energy cohort, the mean time to irrigation and debridement was 3.2 days from injury. Twenty-four patients went to the operating room after ≥2 days from injury, or were treated nonoperatively, and only 1 of these patients had an infection (4.2%). Similarly, 21 patients were treated with closed reduction and surgery at an outpatient day surgery center. The average time to operative treatment from injury to outpatient surgery was 4.4 days, and only 1 patient treated surgically at an ambulatory outpatient center experienced a postoperative infection (4.8%.)

Our study was consistent with previously published literature for distal radius fractures with respect to the incidence of open distal radius fractures and associated complications. Rundgren et al^2^ in a national registry study of 31,807 patients treated surgically for distal radius fracture, found an overall incidence of surgical site infection of 10% among all patients, where the reported incidence of open fractures was 2%. We identified 4 infections in 55 fractures with an overall infection rate of 7.3%, a number comparable to registry data which predominately consists of closed injuries. Glueck et al^13^ found a similar infection rate of 7% in 42 open fractures. While surgical site infection rate after operative fixation is variable, our study of open distal radius fractures had a similar incidence of postoperative infection when compared to the surgical site infections in a national registry of all distal radius fractures,^2^ as well as studies of open distal radius fractures. This speaks to the generalizability of our findings and its applicability in informing appropriate treatment of these injuries.

We found all infections occurred in high energy open fractures. High energy injuries would be expected to be concomitant with more considerable soft tissue injury, subsequent necrosis, and infection. This is consistent with published work showing higher energy mechanisms of injury for open distal radius fractures at a level 1 trauma center were associated with increased complication rates.^18^ These findings also may support the hypothesis of previous investigators that high degree of soft tissue disruption and gross wound contamination may be a more important factor in infection than time to operative debridement.^4^^,^^13^ Higher energy injuries are associated with greater fracture and soft tissue displacement at the time of injury, along with deeper penetration of foreign debris and micro-organisms, contributing to the risk of postoperative infection. This cohort appears to have over a third of patients who underwent temporizing fixation in the form of external fixation or dorsal spanning plate as their initial procedure. When these patients were investigated further, nearly two-thirds of them had high energy mechanisms. This may be a result of the disproportionate amount of high energy, complex trauma seen at the institutions in this cohort. Nevertheless, one might interpret this as making the results found in this study more generalizable to the community, given that even in this complex cohort certain patients were able to undergo outpatient surgery without an increased rate of infection.

In our study, 21 patients were treated surgically in ambulatory surgery centers after being reduced in the emergency department and discharged, with an additional 3 treated nonoperatively. Within this ambulatory cohort there was only 1 infection. Safe triage of isolated, low energy distal radius fractures to outpatient settings in ambulatory patients may lower costs and allow orthopedic surgeons to help alleviate critical inpatient acute medical care shortages responsibly.^19^^,^^20^

This study must be interpreted with an understanding of its limitations. One limitation is that it includes a relatively small sample size of 55 open distal radius fractures. However, open distal radius fractures are a relatively rare injury, as are postoperative infection, and our study has a comparable number of patients to other published work on this topic.^9^^,^^12^^,^^13^ This is a retrospective study and, therefore, is subject to the inherent challenges of retrospective chart review, including incomplete documentation and the potential for differing operative preferences between surgeons from the two contributing institutions. Additionally, given its retrospective nature, the specifics of how patients in this cohort were triaged is not defined as clearly as they would be if it was prospective. In general, gross contamination, larger wounds, and subsequent higher Gustilo Anderson grades were taken to the operative room urgently. In contrast, low energy, type 1 injuries made up a larger percentage of those patients who were sent out of the emergency department. Other possible confounding factors, such as polytraumatized patients, disposition challenges, and patient preference, may have impacted the triage decisions and is a limitation of these data. This study does include patients from 2 different level 1 trauma centers, in separate hospital networks, which makes the results more generalizable.

This study evaluates a cohort of patients with open distal radius fractures and shows that even without urgent irrigation and debridement, the infection rate is comparable to those reported in a national registry data of predominantly closed injuries.^2^ All infections in this study occurred in patients with high energy mechanisms who had at least 1 additional risk factor, such as smoking or type ≥2 open injury. This suggests that urgent operative irrigation and debridement may not be necessary in type 1 open, low energy distal radius fractures without gross contamination. This may allow triage of patients with these injuries to receive care in an ambulatory setting after appropriate reduction and emergency room management, improving health care use and costs. Further prospective studies controlling for mechanism of injury and gross contamination would better clarify the effect of surgical timing on infection following open distal radius fractures.

## Conflicts of Interest

No benefits in any form have been received or will be received related directly to this article.
